# Supplementary material for: Magnetic cilia carpets with programmable metachronal waves
Source: Nat Commun. 2020 May 26;11:2637. doi: 10.1038/s41467-020-16458-4 (PMC7250860; doi:10.1038/s41467-020-16458-4)
Supplement: Supplementary file 1 — Supplementary Information [file 41467_2020_16458_MOESM1_ESM.pdf]

Supplementary Information

Magnetic Cilia Carpets with Programmable Metachronal Waves

Hongri Gu[1], Quentin Boehler[1], Haoyang Cui[1], Eleonora Secchi[2], Giovanni Savorana[2], Carmela De Marco[1], Simone Gervasoni[1], Quentin Peyron[3,4], Tian-Yun Huang[1], Salvador Pane[1], Ann Hirt[5], Daniel Ahmed[1], Bradley J. Nelson[1*]

1. *Institute of Robotics and Intelligent System, ETH Zurich, 8092 Zurich, Switzerland;*
2. *Institute of Environmental Engineering, ETH Zurich, 8093 Zurich, Switzerland;*
3. *ICube Lab, UDS-CNRS-INSA, Illkirch-Graffenstaden 67400, France;*
4. *FEMTO-ST Institute, Université Bourgogne, Franche Comte, CNRS, Besançon 25000, France;*
5. *Institute of Geophysics, ETH Zurich, 8092 Zurich, Switzerland;*

[*] Correspondence and requests for material should be addressed to B.J.N. (email: bnelson@ethz.ch).

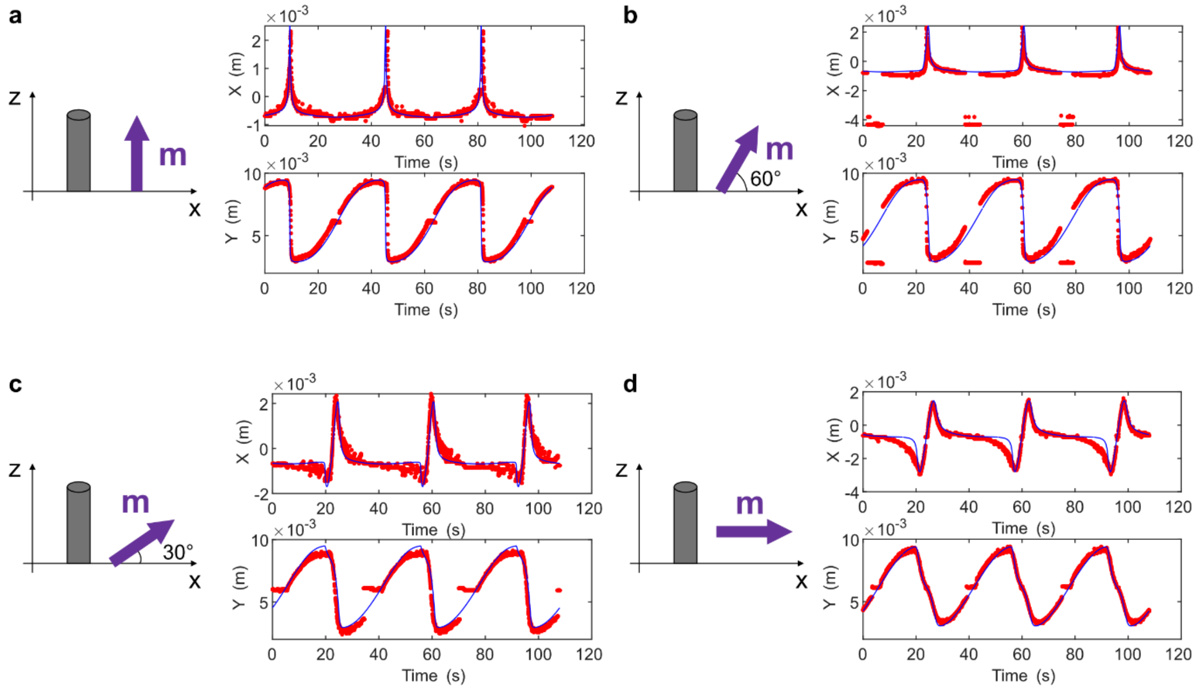

Supplementary Figure 1 | Artificial cilia's tip trajectories of the simulation results (blue curve) and experiments (red dots) for different magnetization directions. All experiments and simulations were performed under a rotating magnetic field (10 degrees per second) of 80 mT inside the x-z plane. The magnetization directions are shown with large purple arrows. Unmatched data, where the tracked tip positions (red dots) are far from simulations (blue curve), is due to the limited tracking algorithm.

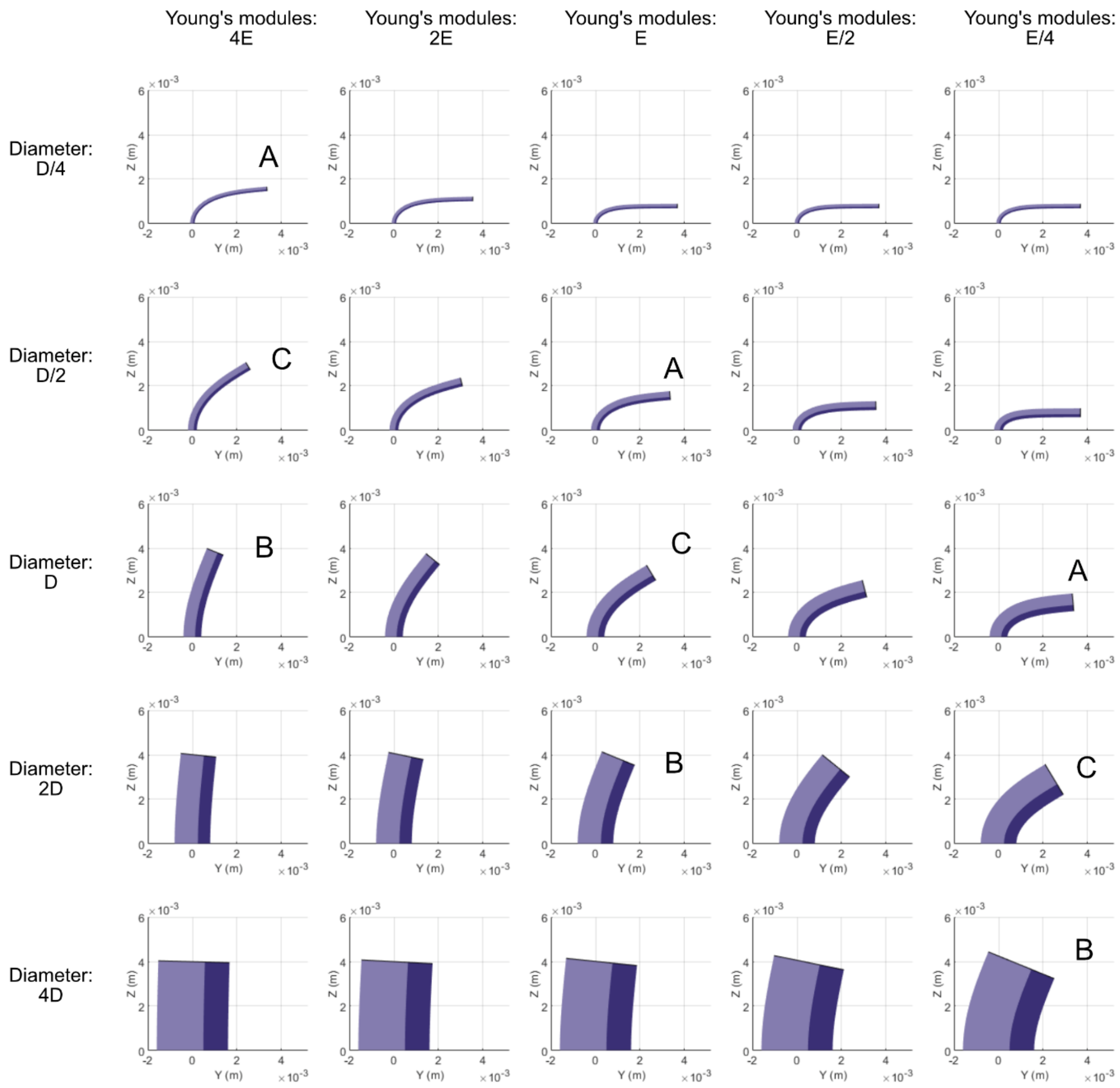

Supplementary Figure 2 | Simulation results of cilia hair bending with different aspect ratios and different stiffnesses. In the simulations we vary the diameter D=0.8 mm of the cylindrical hair and the Young's Modulus $E=1.85\times10^5$ Pa.

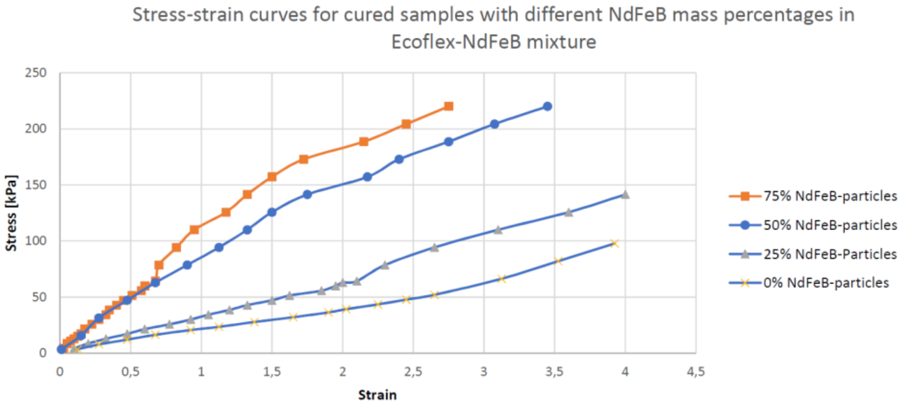

Supplementary Figure 3 | Mechanical characterizations (engineering stress-strain curves) of the Ecoflex-NdFeB composite materials with different weight fractions of the NdFeB microparticles.

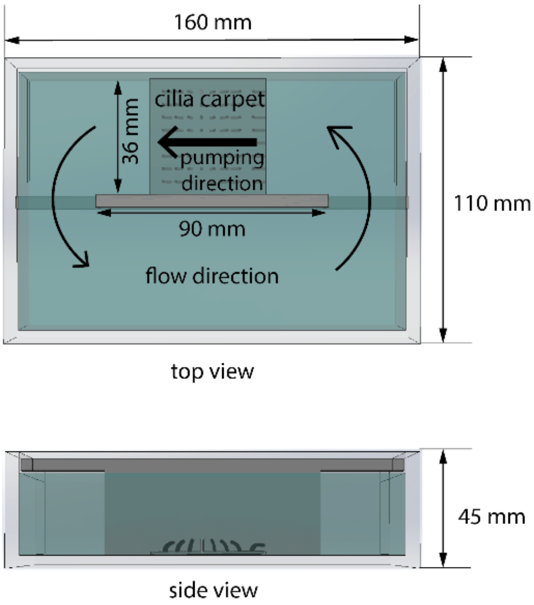

Supplementary Figure 4 | Top and side views of the fluidic setup. The cilia carpet is placed in a 160 mm large, 110 wide and 45 mm deep acrylic box. The observation window is the 160 mm large side of the box. In the middle width of the box (55 mm from the observation window), we place a 90 mm large and 45 mm high white Polypropylene (POM) board. The square cilia carpet (36 mm × 36 mm) is placed between the observation window and the POM board. The box is filled with 99% glycerol (viscosity: 1.15 Pa·s at 20°C) up to a height about 30 mm.

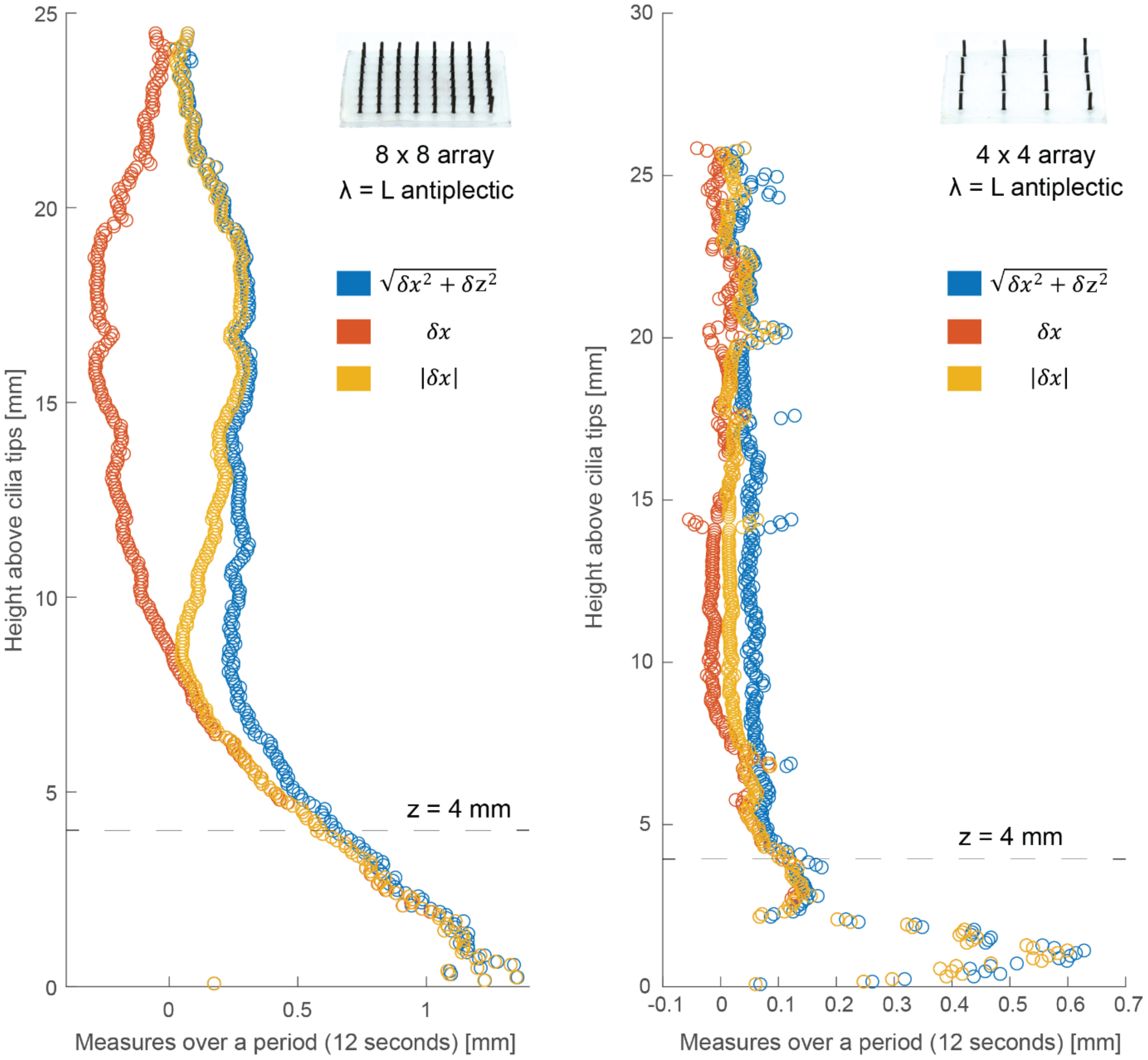

Supplementary Figure 5 | Comparison of different measures to quantify the flow in the x-direction. The 8x8 cilia array with antiplectic wave is shown, on the left, as a high-speed flow example. The 4x4 cilia array with antiplectic wave is shown, on the right, as a low-speed flow example. Both cases show that different measures of the transport in the region of [0, 4 mm] are equivalent to a good approximation. Blue circles are the displacement magnitude $\sqrt{\delta x^2 + \delta y^2}$, as shown in Figure 3c and Figure 4d in the manuscript. Red and yellow circles represent the projections of the displacement vector in x-direction, δx, and its absolute value $|\delta x|$, respectively.

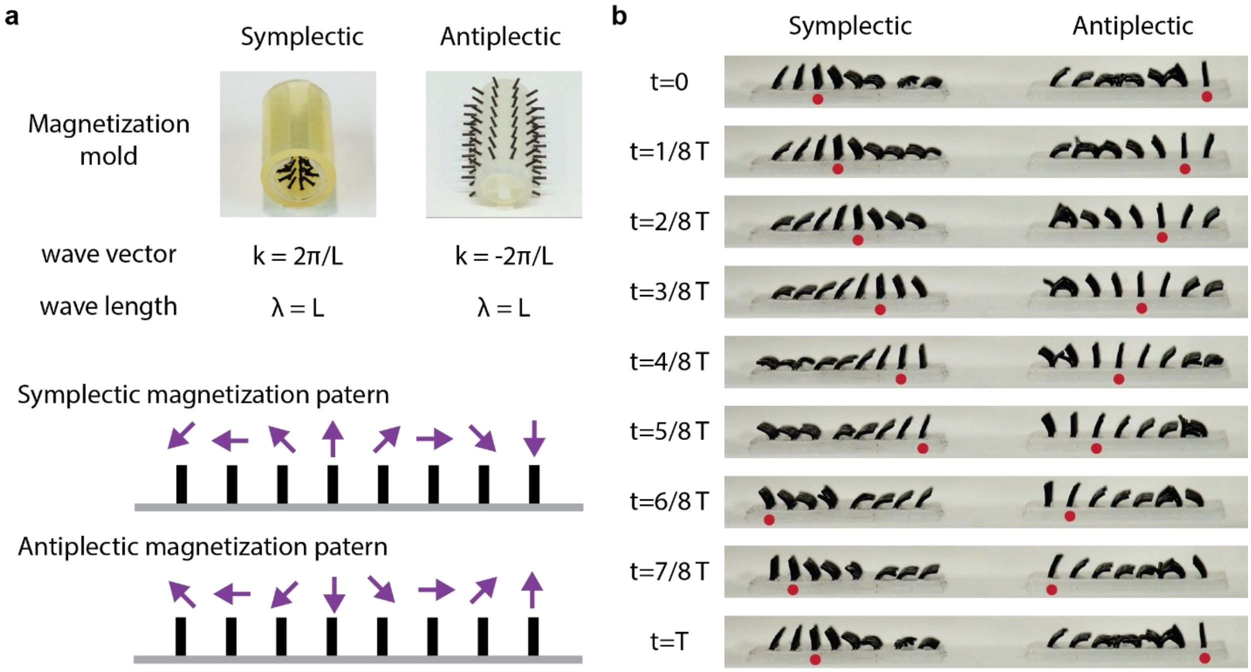

Supplementary Figure 6 | Comparison between symplectic and antiplectic waves on the 8x8 magnetic artificial cilia carpets. a, Encoded symplectic and antiplectic waves on the cilia carpets. Magnetization direction of individual hair are illustrated by the purple arrow. **b**, Snapshots of the 8x8 cilia carpet under one period of rotating magnetic field of 80 mT at 30 degrees per second clockwise in x-z plane. The red dots represent the straight hair (magnetization direction matches the external magnetic field) in the current frame, as a marker to identify the wave traveling direction.

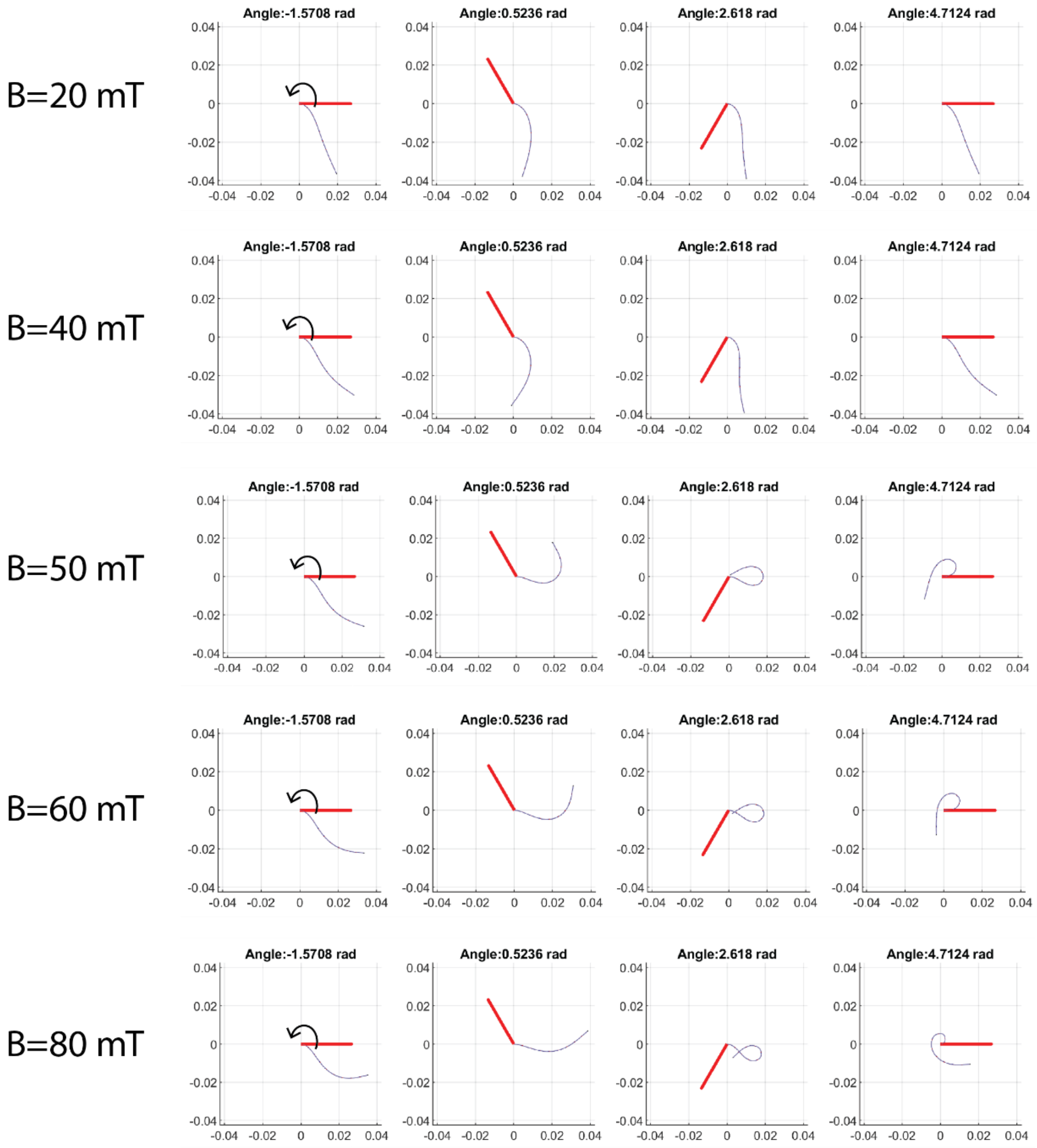

Supplementary Figure 7 | Simulations of the transition of rolling and crawling. we use a simplified 2D simulation to show this transition of the soft cilia carpet with encoded magnetization patterns. In the simulation, we consider gravity, elastic bending torque from the soft carpet, and magnetic torques and forces. The carpet is confined in a two-dimensional plane with one fixed end (position and angle). We then simulate 360-degrees counterclockwise rotating magnetic field with different magnitude, under quasi-static conditions. The carpet will not rotate if the magnetic field is too weak (20 and 40 mT), and the carpet will roll up if the magnetic field is strong enough to overcome gravity and create a roll (superior or equal to 50 mT in this case). The magnetic field directions are indicated by the red lines.

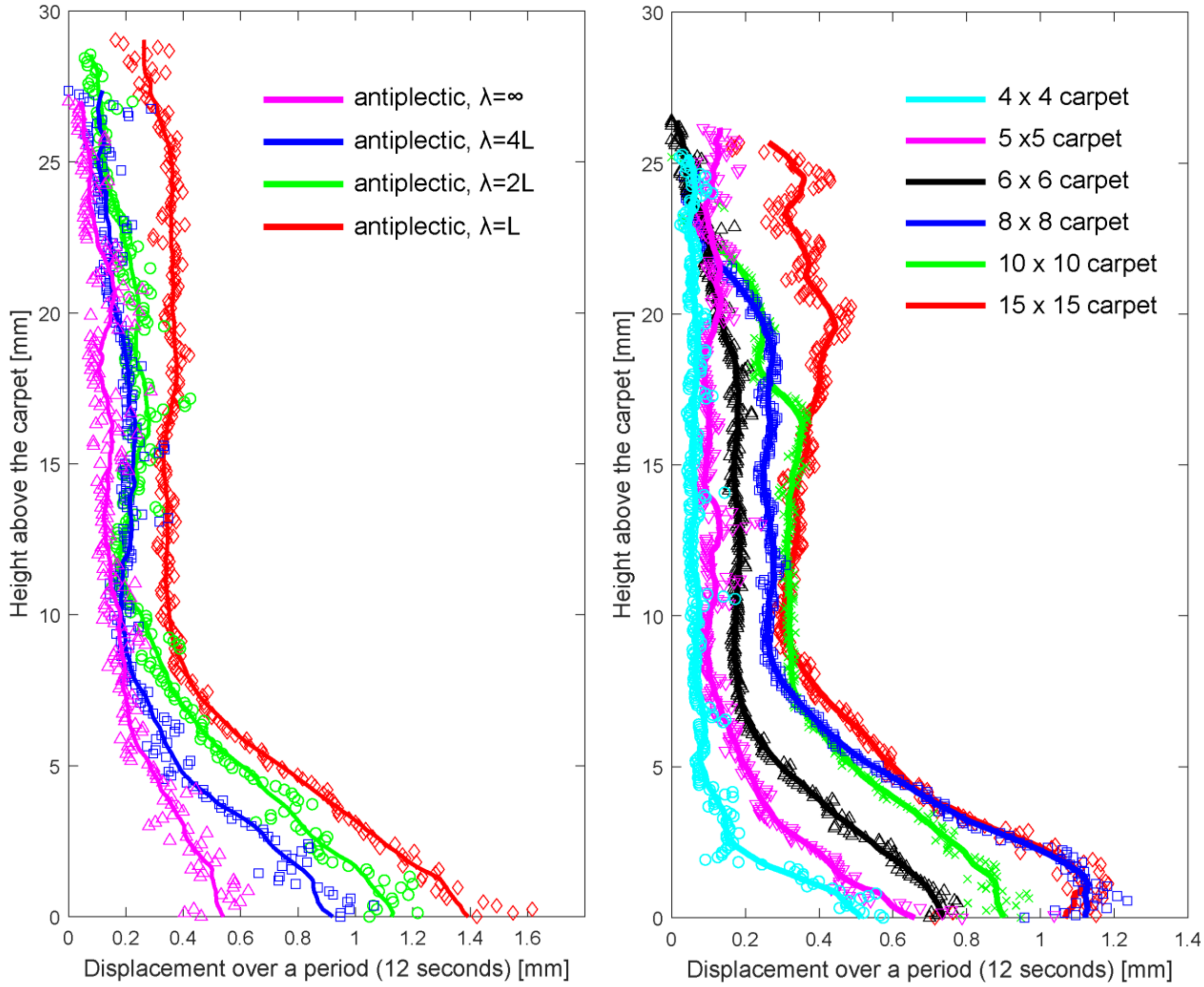

Supplementary Figure 8 | Average displacement over a period from the top of the cilia carpet to the air water interface. The first 15 mm are also reported in Figure 4c (left panel) and 5d (right panel).

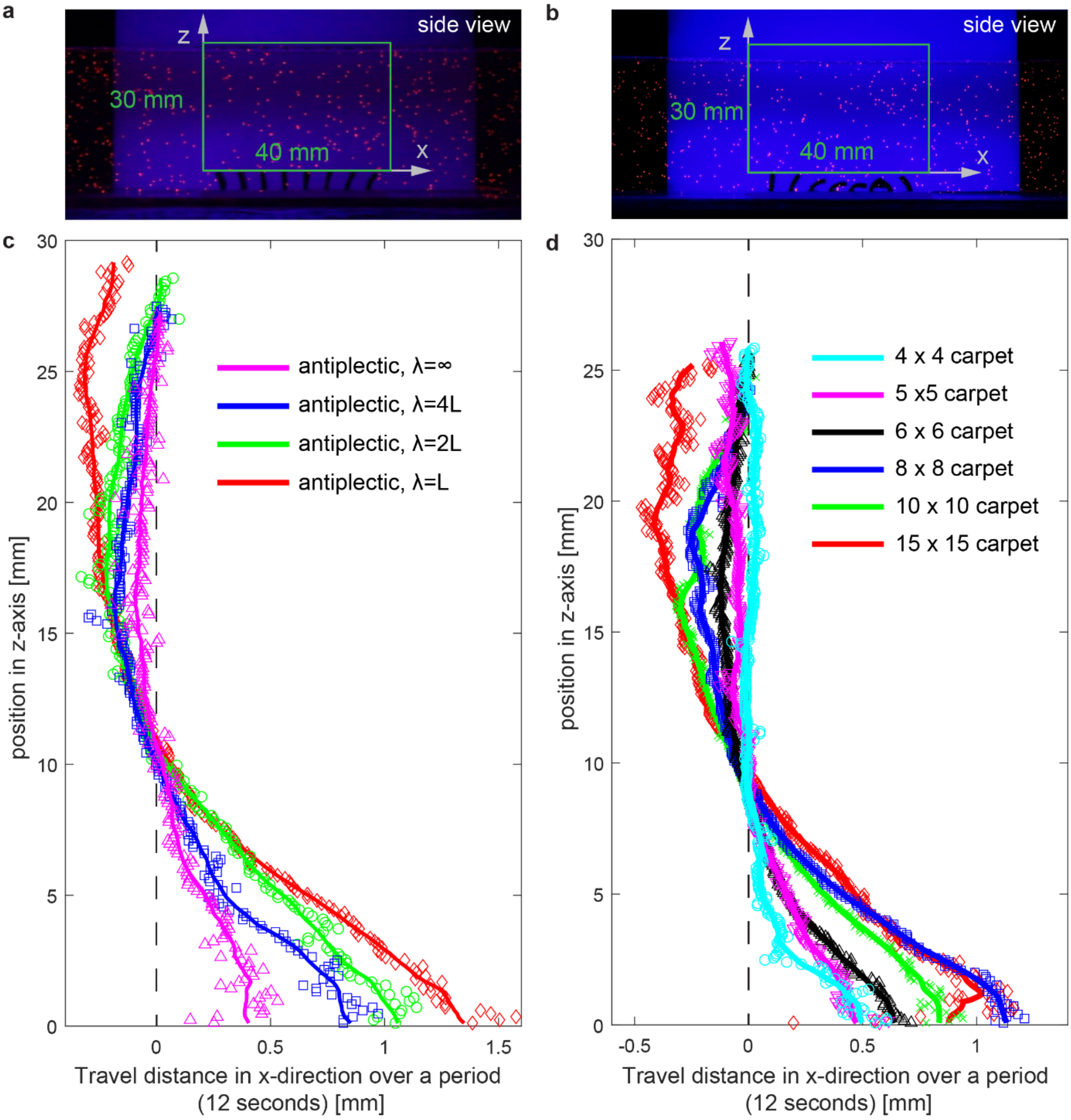

Supplementary Figure 9 | The averaged projections of displacement vectors in x-direction of the fluorescent particles. (**a**,**b**) The tracking region (depicted as the green box) of the tracer particles in the pumping experiments. (**c**) The displacement in the x-direction over a period of 8x8 cilia carpets with various metachronal wavelengths. The magnitude of the displacement vectors of the same experiments is shown in Figure 4c in the manuscript. (**d**) The displacement in the x-direction of cilia carpets with different cilia densities over a period. The magnitude of the displacement vectors of the same experiments is shown in Figure 5d in the manuscript. The colored lines in panel c and d are smoothed results of nearest 21 points.

reference	Cilia length L (μm)	Near carpet flow speed v (μm/s)	Frequency f (Hz)	Normalized speed v/fL
[1]	30	4	0.5	0.27
[2]	500	500	7	0.143
[3]	350	75	10	0.0214
[4]	25	9	34	0.011
This work	4000	83	0.083	0.25

Supplementary Table 1 | Comparison of liquid pumping speed using artificial cilia carpet. The normalized pumping speed v/fL, shows how fast the flow it can generate by one beating cycle.

Supplementary References

1. Vilfan, M. *et al.* Self-assembled artificial cilia. *Proc. Natl. Acad. Sci.* **107**, 1844–1847 (2010).
2. Rockenbach, A. & Schnakenberg, U. The influence of flap inclination angle on fluid transport at ciliated walls. *J. Micromechanics Microengineering* **27**, 015007 (2017).
3. Zhang, S., Wang, Y., Lavrijsen, R., Onck, P. R. & den Toonder, J. M. J. Versatile microfluidic flow generated by moulded magnetic artificial cilia. *Sensors Actuators B Chem.* **263**, 614–624 (2018).
4. Shields, a R. *et al.* Biomimetic cilia arrays generate simultaneous pumping and mixing regimes. *Proc. Natl. Acad. Sci.* **107**, 15670–15675 (2010).
