# Supplementary material for: Magnetic cilia carpets with programmable metachronal waves
Source: Nat Commun. 2020 May 26;11:2637. doi: 10.1038/s41467-020-16458-4 (PMC7250860; doi:10.1038/s41467-020-16458-4)
Supplement: Supplementary file 3 — Description of Additional Supplementary Files [file 41467_2020_16458_MOESM3_ESM.pdf]

Description of Additional Supplementary Files

Supplementary Movie 1: Metachronal waves with different wavelengths

Supplementary Movie 2: Single cilium simulation and characterizations

Supplementary Movie 3: Changing wave directions on a single cilia carpet

Supplementary Movie 4: Fluidic transport on artificial cilia carpets

Supplementary Movie 5: Multimodal locomotion

Supplementary Movie 6: Millipede-inspired soft robot

Supplementary Movie 7: Comparison between symplectic and antiplectic metachronal waves

Supplementary Movie 8: Cilia soft robots walk with different metachronal waves
